# The Anti-Thrombotic Effects of PCSK9 Inhibitors

**DOI:** 10.3390/ph16091197

**Published:** 2023-08-22

**Authors:** Martin Jozef Péč, Jakub Benko, Jakub Jurica, Monika Péčová, Marek Samec, Tatiana Hurtová, Tomáš Bolek, Peter Galajda, Martin Péč, Matej Samoš, Marián Mokáň

**Affiliations:** 1 Department of Internal Medicine I, Jessenius Faculty of Medicine in Martin, Comenius University in Bratislava, 036 59 Martin, Slovakia; pec5@uniba.sk (M.J.P.);; 2 Department of Cardiology, Teaching Hospital Nitra, 949 01 Nitra, Slovakia; 3 Oncology Centre, Teaching Hospital Martin, 036 59 Martin, Slovakia; 4 Department of Hematology and Transfusiology, Jessenius Faculty of Medicine in Martin, Comenius University in Bratislava, 036 59 Martin, Slovakia; 5 Department of Pathological Physiology, Jessenius Faculty of Medicine in Martin, Comenius University in Bratislava, 036 59 Martin, Slovakia; 6 Department of Infectology and Travel Medicine, Jessenius Faculty of Medicine in Martin, Comenius University in Bratislava, 036 59 Martin, Slovakia; 7 Department of Dermatovenerology, Jessenius Faculty of Medicine in Martin, Comenius University in Bratislava, 036 59 Martin, Slovakia; 8 Department of Medical Biology, Jessenius Faculty of Medicine in Martin, Comenius University in Bratislava, 036 59 Martin, Slovakia; 9 Division of Acute and Interventional Cardiology, Department of Cardiology and Angiology II, Mid-Slovakian Institute of Heart and Vessel Diseases (SÚSCCH, a.s.) in Banská Bystrica, 974 01 Banská Bystrica, Slovakia

**Keywords:** LDL, lipoprotein a, platelet activation, thrombus

## Abstract

Atherosclerosis is the primary process that underlies cardiovascular disease. The connection between LDL cholesterol and the formation of atherosclerotic plaques is established by solid evidence. PCSK9 inhibitors have proven to be a valuable and practical resource for lowering the LDL cholesterol of many patients in recent years. Their inhibitory effect on atherosclerosis progression seems to be driven not just by lipid metabolism modification but also by LDL-independent mechanisms. We review the effect of PCSK9 inhibitors on various mechanisms involving platelet activation, inflammation, endothelial dysfunction, and the resultant clot formation. The main effectors of PCSK9 activation of platelets are CD36 receptors, lipoprotein(a), oxidised LDL particles, tissue factor, and factor VIII. Many more molecules are under investigation, and this area of research is growing rapidly.

## 1. Introduction

Despite new therapy options, cardiovascular events remain the most significant cause of morbidity and mortality in western countries. Atherosclerosis is the primary process that underlies cardiovascular disease. The connection between LDL cholesterol and the formation of atherosclerotic plaques has been established by years of research. Low-density lipoproteins (LDL) and high-density lipoproteins (HDL) play a central role in the transport of cholesterol [1]. The function of LDL is to transport about 60% of serum cholesterol from the liver to peripheral tissues, including the arterial walls [2]. An essential part of LDL is apolipoprotein B100 (apoB-100), which permits LDL to attach to its receptor. HDLs, in which the primary protein portion is apolipoprotein A-I, carry cholesterol from peripheral organs to the liver, enabling its elimination. The balance between HDL and LDL, and their synthesis and elimination, is essential for regulating the amount of circulating cholesterol. More than 70% of LDL is eliminated from the bloodstream by attaching to the LDL receptor in the liver [3]. Both European and American guidelines recommend a rigorous reduction in LDL cholesterol. A low LDL concentration is related to a reduction in cardiovascular events in primary and secondary prevention.

LDL cholesterol, apoB-100, and plasminogen-like apolipoprotein(a) make up the plasma protein lipoprotein(a), which is another modifiable risk factor linked to cardiovascular events [4]. LDL cholesterol and lipoprotein enter the arterial wall and are accumulated there. Lp(a) is reliant on Lp(a) plasma concentrations, Lp(a) particle size, blood pressure, and artery wall permeability. LDL cholesterol reaches the intima via the LDL receptor [5]. Both are absorbed by macrophages, which create foam cells and aid in forming atherosclerotic plaques [6].

**Figure 1:**
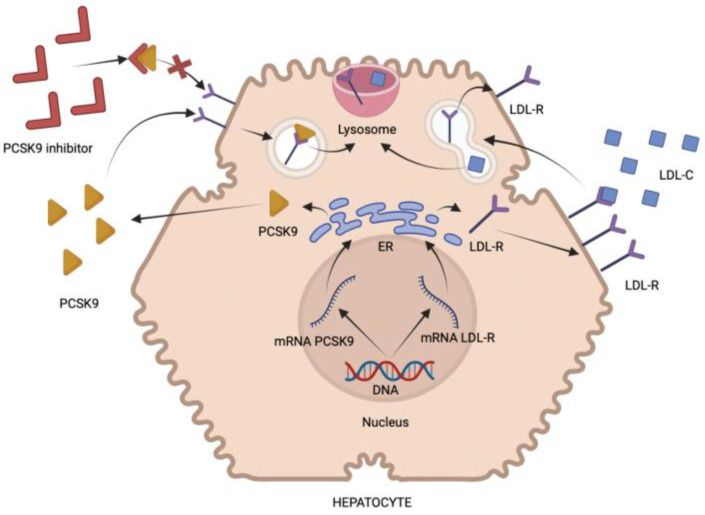
Figure illustrating the impact of PCSK9 inhibitors on the LDL-dependent pathway. Low-density lipoprotein receptors (LDL-R) are produced in the hepatocyte and subsequently are transported to the cell membrane. Low-density lipoprotein (LDL) particles bind to LDL-R and then they form a complex with LDL, which is endocytosed. The LDL is degraded, and LDL-R is transported back to the membrane. Moreover, PCSK9, produced in the endoplasmic reticulum (ER), binds to LDL-R and forms a PCSK9-LDL-R complex, which undergoes endocytosis and lysosomal degradation. The inhibitors of PCSK9 bind to free PCSK9 molecules and prevent them from creating a PCSK9-LDL-R complex and their subsequent degradation.

The elimination of LDL is carried out by LDL receptors (LDLR). After binding to LDLR, the LDL part of the complex is degraded by lysosomal enzymes, and the receptor is used again on the cell membrane to bind to another LDL [7]. Lower concentrations of intracellular cholesterol result in a higher amount of surface LDL receptors on the hepatocyte membrane [8,9]. Another path for the regulation of LDL receptors is via proprotein convertase subtilisin/kexin type-9 (PCSK9). The liver is vital for the synthesis of this protein, but it is also produced in the kidney, brain, pancreas, and intestine. The role of PCSK9 is to prevent LDL receptor degradation, resulting in a reduced amount of LDLR on the cell surface [10] (Figure 1). LDL-reducing therapy is critical for a reduction in cardiovascular risk. Interest in influencing PCSK9 was increased after the finding that the excessive expression of PCSK9 in mice caused a significant increase in plasma cholesterol [11].

Inhibiting PCSK9 expression decreases serum LDL concentrations and the risk of cardiovascular disease. According to a meta-analysis of a large patient cohort, a significant positive correlation between circulating PCSK9 concentrations and the risk of major adverse cardiovascular events was found [12].

**Figure 2:**
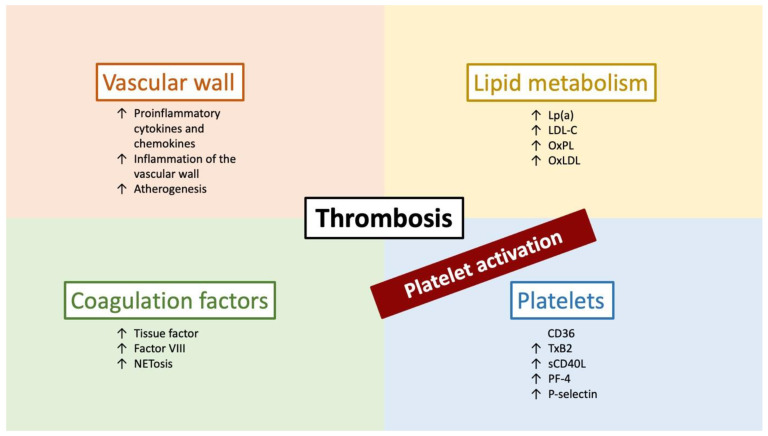
Effect of PCSK9 on thrombosis mediated through different mechanisms.

In recent years, it was shown that lipid-lowering drugs have some effects unrelated to lipid metabolism [13,14]. It appears that PCSK9 inhibitors also influence platelet function, inflammation, and endothelial dysfunction. This study aims to provide a narrative review of the possible anti-thrombotic effects of PCSK9 inhibitors (Figure 2).

## 2. Methods

This article is designed as a non-systematic (narrative) review. In an effort to meet these requirements, we performed a search of the three most important medical literature databases—PubMed Central, Scopus and Web of Science. We used keywords related to the topics—“PCSK9 inhibitors” or “proprotein convertase subtilisin/kexin type-9” and “thrombosis” or “venous thromboembolism” or “anti-thrombotic effect” to identify relevant articles from the recent medical literature. Afterwards, the results were reviewed by the authors and this narrative review article summarises the most recent and relevant data.

## 3. Proprotein Convertase Subtilis Kexin-9

Since its discovery in 2003, pro-protein convertase subtilisin/kexin type 9 (PCSK9), a soluble protease, has been the subject of a significant amount of research in the fields of cholesterol homeostasis and cardiovascular biology [15]. PCSK9, previously named Neural Apoptosis Regulated Convertase 1 (NARC1), is a member of the Pro-protein Convertases family of secretory serine proteinases [16]. PCSK9 is mainly produced in the hepatocytes of the liver and is subsequently released into circulation. The hepatocyte is not the only cell that can produce and secrete PCSK9; the cells of the intestines [17,18], adipose tissue [19], pancreas [20], brain [21] and kidneys [22] also have this ability. It is interesting to note that PCSK9 biologically fluctuates throughout the day, with late-night levels rising and late-afternoon levels falling [23]. Additionally, females have higher overall circulating PCSK9 levels than males, indicating that hormones like oestrogens are involved in the expression and secretion of PCSK9 [24]. A recent study showed that PCSK9 inhibitors are less effective at reducing LDL-c levels in women than in men, implying the possible effect of oestrogens and the importance of sex differences in the metabolism of PCSK9 [25]. It was found that multiple metabolic factors influence the levels of PCSK9, including plasma cholesterol and triglyceride levels, blood pressure, age, and body mass index [26]. The 22 kb human PCSK9 gene is found on chromosome 1p32 [16]. A 692 amino acid proteinase is encoded by its 12 exons and 11 introns [16]. PCSK9 is released in a non-active form and subsequently undergoes posttranslational modification to become a mature protein [27]. PreProPCSK9 contains five parts, but the most important is the C-terminal domain, where its M2 part is located. The M2 part plays an important role in forming the PCSK9-LDLR complex [28].

## 4. Platelet Activation Markers and PCSK9 Inhibitors

Recent studies have proved that PCSK9 can potentially modify platelet function. Through a variety of methods, dyslipidaemia can affect haemostasis and platelet reactivity. High LDL cholesterol concentrations relate to increased platelet reactivity and the elevated production of thromboxane. Oxidised LDL is formed because of the increased oxidative stress associated with high concentrations of circulating LDL cholesterol, significantly contributing to inflammation-driven thrombosis [29]. Oxidised LDL is the primary factor that increases platelet activation via CD36. PCSK9 binds to CD36, a negative angiogenesis regulator [30]. By identifying specific oxidised phospholipids and lipoproteins, CD36 is involved in the internalisation of apoptotic cells, bacterial and fungal infections, and LDL, triggering inflammatory reactions that alter the development of atherosclerosis. PCSK9 activates platelets after binding to CD36, and inhibitors of PCSK9 are decreasing their activity. The PCSK9—REACT study studied the relationship between PCSK9 concentrations and platelet activation. The conclusion was that increased PCSK9 is connected to platelet activation, and PCSK9 should be considered a predictor of ischemic events [31]. A recent study from Qi et al. showed that by binding to platelet receptor CD36 and activating the downstream signalling pathways, PCSK9 in the plasma directly increases platelet activation and in vivo thrombosis [32]. Platelet activation, soluble Nox2-derivated peptide, and oxidised LDL were monitored in patients with familial hypercholesterolemia before, and after, treatment with PCSK9 inhibitors. The results revealed decreased LDL, oxidised LDL, and PCSK9 concentrations [33]. Authors Cammisotto et al. proved that PCSK9 directly interacts with platelets through the CD36 receptor and activates Nox2. They showed that LDL amplifies this effect [34]. Soluble P-selectin and soluble CD40 ligands, the markers of platelet activation connected to cardiovascular risk, decreased after treatment with 10-Dehydragingerdione, which reduced PCSK9 production [35].

Recent research has demonstrated that the plasma protein PCSK9 interacts with the CD36 receptor and activates the ROS-upregulating enzymes Src kinase, mitogen-activated protein kinase (MAPK), extracellular signal-regulated kinase 5, and C-Jun amino-terminal kinase. Additionally, activating the CD36-dependent p38MAPK/cytoplasmic phospholipase A2/cysin-1/thromboxane A2 signalling pathway promoted platelet activation and thrombosis in vivo [32]. Some studies suggest that PCSK9 also plays a role in regulating CD36 and triglyceride metabolism [36]. By lowering Lp(a) concentrations, PCSK9 inhibitors may lessen the direct and indirect stimulatory effects of Lp(a) on platelets [37]. Marston et al., in their meta-analysis of the FOURIER and ODYSSEY OUTCOMES studies, demonstrated a 31% reduction in venous thromboembolism relative risk in patients treated with PCSK9 inhibitors. The reduction in this risk was associated with Lp(a) baseline concentrations, and the authors suggested Lp(a) as a marker for observation [38]. In another study, D-dimer and fibrinogen concentration changes were not observed in patients with familial hypercholesterolemia. On the other hand, a reduction in plasma PAI-1 levels was documented [39,40]. Additionally, PCSK9 inhibitors raised HDL cholesterol concentrations [41], which may prevent platelet aggregation directly or indirectly by lowering platelet membrane cholesterol [42]. Finally, PCSK9 was shown to increase apoptosis in vascular smooth muscle and endothelial cells. Cell death is known to favour thrombosis, partly through the production of procoagulant microparticles. In this situation, PCSK9 inhibitors may indirectly prevent thrombosis by reducing apoptosis [43,44,45]. Additionally, it was recently established that platelets secrete PCSK9 after activation in the presence of LDL, further increasing the platelets’ ability to aggregate and form thrombi. Furthermore, this encourages the conversion of monocytes into macrophages and foam cells, which also contributes to atherogenesis [46,47].

The impact of PCSK9 inhibitors on platelet activity highlights the interaction between PCSK9 and platelet function modification in humans. A study by Barale et al. [48] showed that platelet activation markers—sCD40L, platelet factor 4, and sP-selectin— significantly decreased after treatment with PCSK9 inhibitors. These markers correlate with PCSK9. Studies summarising the non-lipid effects of PCSK9 and PCSK9 inhibitors, mainly on the platelet activation markers, are listed in Table 1.

As mentioned above, PCSK9 levels have a positive correlation with platelet activation markers such as sCD40L, platelet factor 4, and sP-selectin. In order to predict major adverse cardiovascular events (MACEs) in coronary artery disease (CAD), circulating PCSK9 levels were proposed as a new biomarker [55]. In stable CAD patients with diabetes mellitus (DM), the studies found that baseline PCSK9 levels were independently related to the risk of MACEs. Patients with high PCSK9 levels with DM had a significantly higher risk of MACEs than those with low PCSK9 levels and non-DM [56]. These results imply that measuring plasma PCSK9 could aid in identifying diabetic people with CAD who are at higher cardiovascular risk. According to the study by authors Song et al., STEMI patients with DM undergoing primary percutaneous coronary intervention (PCI) who had high circulating PCSK9 levels were at an elevated risk of MACEs. The elevated risk of severe cardiovascular events in patients with myocardial infarction with ST segment elevation (STEMI) with high PCSK9 levels and DM may be due to the substantial association between PCSK9 and indicators of inflammation and platelet activation. The results of this study point to a possible advantage of PCSK9 inhibition in the early stages of acute coronary syndrome (ACS), particularly for patients with DM and high PCSK9 levels, by a dual mechanism on both lipid-lowering and inflammation/platelet pathways [57].

## 5. Endothelial Dysfunction Markers and PCSK9 Inhibitors

The endothelium plays a crucial role in maintaining vascular homeostasis [58]. One of the earliest signs of atherosclerosis, endothelial dysfunction, contributes to the development of plaques and the problems associated with atherosclerosis. The leading causes of endothelial dysfunction are cardiovascular risk factors (e.g., cholesterol concentration) and oxidative stress [59].

Several studies have shown the effect of PCSK9 inhibitors on endothelial function and the correlation between PCSK9 and endothelial dysfunction. El-Seweidy et al. studied endothelial dysfunction markers in addition to PCSK9 in a rabbit model. Their study showed a correlation between PCSK9 suppression and atherogenic and coronary risk index reduction [35]. The study from authors Maulucci et al. proved that treatment with a PCSK9 inhibitor improved endothelial function measured by mean brachial artery diameter, velocity time integral, and flow-mediated dilation (FMD) [60]. Another study used magnetic resonance imaging technology to noninvasively measure coronary endothelial function in patients living with human immunodeficiency virus infection and those with dyslipidaemia. Before PCSK9 inhibitor treatment, there was a decrease in, or no dilatation of, coronary arteries and no improvement in coronary blood flow. After treatment, the patients showed better outcomes in MRI monitoring of coronary endothelial dysfunction and increased coronary blood flow, representing an improvement in coronary artery health [61]. In vivo and ex vivo analyses proved that PCSK9 inhibitors impair endothelial dysfunction by limiting interactions among leukocytes and endothelium [54]. A possible pleiotropic effect of PCSK9 inhibitors can be found in connection to circulating endothelial progenitor cells (cEPCs). A recent study by authors Itzhaki Ben Zadok et al. on a cohort of 26 patients showed a significant increase in EPCs and VEGF after PCSK9 inhibitor treatment. Higher numbers of active cEPCs were seen in patients with CVD receiving PCSK9 inhibitor treatment, demonstrating the stimulation of endothelium repair. These results could indicate a brand-new PCSK9 inhibitor mode of action [62]. Treatment with inhibitors of PCSK9 increases cEPCs activity and differentiation into endothelial cells.

The septic state is often connected with dysfunction of the endothelium. According to a recent study, sepsis-related elevated PCSK9 expression triggered the TLR4/MyD88/NF-B and NLRP3 pathways to cause inflammation, leading to vascular endothelial dysfunction and lowered survival rates. Improved vascular endothelial function in sepsis may be achieved through the clinical therapeutic target of PCSK9 inhibition [63].

The last randomised active-controlled trial proved the cumulative benefit of adding PCSK9 inhibitors to sodium-glucose cotransporter 2 inhibitors (SGLT2i). SGLT2i are known for impairing endothelial dysfunction. Adding a PCSK9 inhibitor to SGLT2i treatment improved endothelial function assessed by FMD and the plasma concentrations of nitrate, nitrite, and isoprostane [64]. PCSK9 drugs suppress endothelial cell proinflammatory activation and reduce apoptosis in endothelial cells, smooth muscle cells, and macrophages [65]. Alirocumab, a PCSK9 inhibitor, also decreased atherosclerotic plaque vulnerability-affecting variables by a significant amount [66].

Di Minno et al. enrolled 25 patients with familial hypercholesterolemia and observed improved endothelial function after 12 weeks of PCSK9 inhibitor treatment in the administration regime every 14 days. Changes in FMD, and in the reactive hyperaemia index, were seen after 12 weeks, and the LDL score change was an independent predictor of the FMD change [67]. The ALIROCKS trial studied 24 patients before, and after, 10 weeks of treatment with a PCSK9 inhibitor. Primary outcomes of the trial were a change in carotid vessel wall fractional anisotropy, a novel magnetic resonance-based measure of vascular integrity, and a change in the carotid intima-media thickness and flow-dependent dilatation of the brachial artery measured by ultrasound. The result was that vascular endothelial growth factors and P-selectin did not alter. Although flow-dependent dilatation was somewhat improved, there were no discernible long-term benefits of PCSK9 inhibitor therapy on vascular function [68]. A specific analysis of the ODYSSEY Outcomes trial (*n* = 18,924 patients with acute coronary syndromes) showed a reduction in major advanced cardiovascular events (MACE). The risk of MACE after recent ACS was predicted by baseline lipoprotein(a) and corrected LDL-C concentrations and their decreases after PCSK9 inhibitor treatment. Alirocumab’s lowering of lipoprotein(a) is an independent factor in decreases in MACE, which implies that lipoprotein(a) should be a separate therapy goal following ACS [69].

In addition, compared to placebo, therapy with PSCK9 inhibitors directly decreased carotid arterial wall inflammation and reduced the volume of atherosclerotic plaques [70]. Importantly, this inflammation reduction was independent of inflammatory markers [71]. The precise mechanism through which the PCSK9 inhibitor enhances endothelial function is unknown. It is probable that a drop in LDL cholesterol concentrations mainly mediates its impact, but other pathways may also have an equal impact.

A vital component in controlling vascular function is the endothelium, which is affixed to the inside surface of blood vessels [72]. The endothelium controls the tensile strength of blood vessel walls, prevents blood from seeping into tissues, and lessens inflammation [73]. Nitric oxide (NO) is created by endothelial nitric oxide synthase (eNOS) when it is joined to its co-factor, tetrahydrobiopterin (BH4). The NO, an endothelial vasodilator, is diminished prior to the formation of atherosclerotic plaques. Endothelial dysfunction and atherosclerosis are both thought to be significantly mediated by defects in NO generation or activity. The dysfunction of eNOS is strongly associated with the pathogenesis of arteriosclerosis, hypertension, myocardial infarction, and stroke [73]. The eNOS affects endothelial cell function directly and plays a crucial role in the regulation of vasodilatation [74]. A recent study discovered that inhibiting PCSK9 reversed the decline in eNOS expression brought on by sepsis; the results supported the author’s theory that sepsis-induced endothelium dysfunction enhanced PCSK9 expression [63]. The in vitro study by authors Zulkapli et al. showed that the co-incubation of alirocumab and evolocumab at specific concentrations resulted in an upregulation of the production of the eNOS protein. Only evolocumab caused an increase, although not significant, in eNOS mRNA [75]. Treatment with a PCSK9 inhibitor given to type 2 diabetes (T2D) patients (110 individuals) along with SGLT2i treatment improved FMD and the bioavailability of NO. Increased NO may contribute towards improving endothelial dysfunction [64].

## 6. Inflammation Markers and PCSK9 Inhibitors

In contrast with statins, PCSK9 inhibitors do not reduce concentrations of high-sensitive CRP (hs-CRP), as demonstrated by several studies and a meta-analysis [76,77]. The same results were obtained regarding concentrations of TNF-α and IL-6 [76,78]. On the other hand, it was established that hs-CRP is positively correlated with PCSK9 concentrations in chronic coronary disease and acute coronary syndrome [58,79]. PCSK9 concentrations are also elevated in patients with systemic inflammatory response syndrome and sepsis [80]. Although hepatocytes are the primary site of PCSK9 production, vascular endothelial and smooth muscle cells express PCSK9 under the regulation of proinflammatory molecules such as IL-1β, TNF-α, and lipopolysaccharide [81]. Therefore, one could argue that, from a clinical perspective, PCSK9 plays more of a role as a by-product of inflammation than as a modulator by itself.

From experimental studies, the potential inflammatory input of PCSK9 could be mediated by the LDLR and the ensuing increased migration of monocytes into the atherosclerotic plaque [82,83]. This was partly presented by a clinical study showing a PCSK9 association with plaque formation independent of LDL concentrations and other traditional risk factors [84]. Another mechanism by which PCSK9 can modulate the migration of monocytes is the chemokine receptor type-2, which was inhibited by PCSK9 inhibitors in patients with familial hypercholesterolemia and decreased the capacity of monocytes to pass the endothelium [85].

It is theorised that PCSK9 upregulates inflammation through the increased expression of TLR-4 and LOX-1, which activate NF-kB and set off the inflammation cascade of other modulators [86]. Another study that evaluated vessel inflammation by PET/CT scanning showed a decrease in the target-to-background ratio after long-term administration of PCSK9 inhibitors [87].

Altogether, there are several theories for the positive LDL-independent effect of PCSK9 inhibition concerning inflammation and following atherothrombosis, but the actual clinical benefit of this data still needs to be evaluated.

## 7. Coagulation Factors and PCSK9 Inhibitors

Research on animals and humans indicates a connection between PCSK9 and thrombotic risk. The animal models showed that after ligation of the inferior vena cava and the subsequent venous thrombosis, PCSK9-/- mice had a comparatively shorter thrombus length, and demonstrated decreased neutrophil extracellular trap (NET) production (NETosis), and leucocyte attachment and accumulation [88]. Leucocyte recruitment was connected to an increased CXCL1. A potential mechanism by which PCSK9 promotes NETosis-induced thrombosis is the recruitment of myeloid cells [39,89]. The study by authors Schuster et al. demonstrated the decreased production of inflammatory molecules after PCSK9 inhibition in mice models [90]. This pathway between PCSK9 and NETosis is one of the possible connections in clot formation.

Levine et al. discovered a downregulation of PCSK9 expression in mice with either pharmacological or genetic PAI-1 inhibition when examining the potential relationship between PCSK9 and fibrinolysis. This was also shown in humans with a mutation that decreased the levels of PAI-1. Additionally, the investigators discovered a significant association between PAI-1 and PCSK9 levels in people with heart failure, pointing to a possible interaction between PCSK9 and the fibrinolytic process [38]. On the other hand, PCSK9 inhibitor treatment decreased PAI-1 levels and increased fibrinolytic activity [91,92].

A higher level of PCSK9 was found in patients at high thrombotic risk with antiphospholipid antibodies [93]. A favorable correlation between PCSK9 levels and fibrinogen levels was also found in patients with stable coronary artery disease [79]; these data show a positive correlation between PCSK9 and the coagulation system [40].

**Figure 3:**
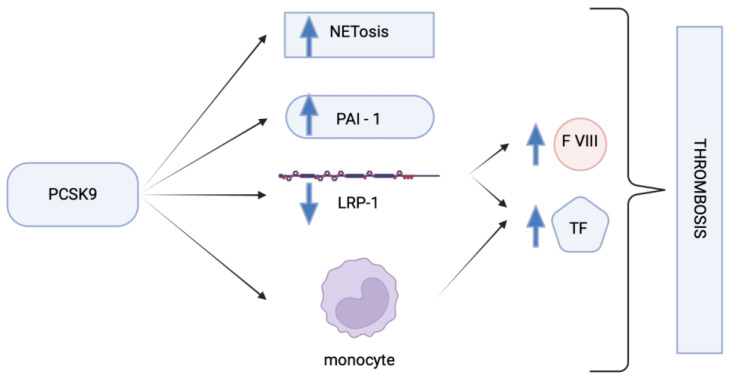
Effect of PCSK9 on coagulation through various mechanisms.

Authors Wang et al. showed a positive association between PCSK9 expression and the levels of tissue factor in patients with coronary artery disease and diabetes [94]. The tissue factor is considered a prothrombotic element, and it is regulated by LDL receptor-related protein-1 (LRP-1) [95]. LRP-1 encourages the degradation of tissue factor. LRP-1 expression is downregulated by PCSK9, which could impact circulating TF levels. Additionally, PCSK9 was shown by authors Scalise et al. to promote TF expression on monocytes, which boost procoagulant activity. The TLR4/NF-B pathway’s activation mediates this mechanism [96]. Both the extracellular vesicle-associated tissue factor and extracellular vesicle formation by procoagulant extracellular vesicles from human mononuclear cells (PBMCs) and THP-1 cells increased due to PCSK9. Pre-treatment with TLR4 (C34) and NF-B signalling (BAY 11-7082) inhibitors reduced the production of extracellular vesicles and extracellularly bound tissue factor triggered by PCSK9. An anti-PCSK9 human monoclonal antibody had a similar effect [97]. In conclusion, PCKS9 can both directly and indirectly boost TF expression (Figure 3).

Through an impact on the blood-clotting factor VIII (FVIII), PCSK9 can also modify blood coagulation [18,98]. An increased arterial and venous thrombosis risk is linked to higher FVIII levels. FVII plays a crucial role in thrombin formation and propagation of the coagulation cascade [99,100]. By facilitating its endocytosis and degradation, LRP-1 inhibits FVIII [101]. This is a potential explanation for how PCSK9 can further contribute to thrombogenesis because it has been demonstrated that it also increases FVIII levels via decreasing LRP-1 expression [102]. It was shown that anti-PCSK9 reduces FVIII levels by enhancing LRP-1 expression in mouse models [103].

## 8. Other Possible Implications of PCSK9 Inhibitors

Overexpression of PCSK9 was found in different cancer types, including colorectal cancer [104], breast cancer [105], lymphoblastic leukaemia [106], hepatocellular carcinoma [107] and gastric cancer [108]. Samples from the cancer regions compared to normal areas showed increased levels of PCSK9 [109]. Additionally, the increased PCSK9 levels were associated with a worse prognosis in many cancers [110]; low levels of PCSK9 are connected to reduced tumour growth and improved prognosis [111]. These results indicate that inhibiting PCSK9 could halt the spread of cancer and suggest the use of PCSK9 as a biomarker of prognosis and response to therapy. The inhibition of PCSK9 caused apoptosis in cancer models in vitro [112,113]. The pathways of the increased apoptosis due to PCSK9 inhibition are mitochondrial signalling pathways and the activation of endoplasmic reticulum stress [114]. The effect of the downregulation is not universal, in a mice model of melanoma, the inhibition of PCSK9 did not demonstrate growth reduction or a better prognosis [115]. On the other hand, in the case of hepatocellular carcinoma, in vivo and in vitro studies proved that PCSK9 has a protective influence against cancer growth and spread [116]. These data imply that, in contrast to the patterns observed with the malignancies previously mentioned, hepatocellular cancer may be an example of a cancer in which the downregulation of PCSK9 may have adverse effects. According to a study from authors Ioannou et al., while PCSK9 overexpression of LDLR on hepatocytes lowered plasma cholesterol and may suppress other malignancies, the increased exposure of the liver to cholesterol may contribute to the development of hepatocarcinogenesis [117]. It was demonstrated that in CD8+ T cells, LDL-R combines with the T-cell receptor (TCR), activating it when it binds to antigenic peptides that the major histocompatibility complex (MHC) presents to tumour cells. The activation of CD8+ T lymphocytes’ antitumor response is increased by the binding of LDL-R to TCR, which encourages cell surface recycling [109].

Viral infections (hepatitis C virus and dengue fever virus) might be associated with PCSK9. Studies identify LDL-R as a binding receptor for the entrance of hepatitis C virus (HCV) into the liver cell [118]. However, virus production is dramatically boosted following LDL-R re-expression, demonstrating that LDL-R is not associated with HCV entry but rather is directly associated with the lipid metabolism of host cells during HCV packaging. Cells lacking functional LDL-R can still become infected with HCV. Patients with HCV should take PCSK9 inhibitors cautiously since they can increase HCV packaging and infectivity [119].

During sepsis, circulating bacterial lipids are assumed to play a crucial role in causing unchecked systemic inflammatory reactions [120]. These lipids, such as LPS, are cleared by LDL-R on the liver cells. PCSK9 inhibitors reduce the density of LDL-R. Based on this knowledge, treatment with PCSK9 inhibitors can represent a new method of sepsis treatment. Indeed, a mice PCSK9-/- model was shown to have lower bacterial numbers in the blood, lungs, and peritoneal fluid than wild-type animals in sepsis models of caecal ligation and perforation, indicating that the deletion of PCSK9 is advantageous for bacterial suppression or clearance [121]. A sub-analysis of the ALBIOS trial study by the authors Vecchié et al. found that patients with septic shock and low plasma levels of PCSK9 had higher mortality rates [122]. A recent study by the authors Zhou et al. did not find a positive or negative connection among PCSK9 inhibitors, sepsis, and severe infections in populations with high cardiovascular risk and does not support the use of PCSK9 inhibitors in preventing sepsis [123].

## 9. Conclusions

The lipid-lowering effect of PCSK9 inhibitors is known and well-documented. PCSK9 stimulates platelets, heightens inflammation, and shifts the balance between fibrinolysis and coagulation in favour of coagulation. PCSK9 inhibitors with evidence of lowering cardiovascular morbidity and mortality decrease Lp(a) levels through unknown processes and reduce LDL cholesterol by boosting the number of LDL receptors. VTE is associated with Lp(a) levels, and the Lp(a) reduction correlates with decreased VTE incidence. It is assumed that PCSK9 alters primary and secondary haemostasis through changing platelet activity, TF, PAI-1, NETosis, and FVIII levels, and through the effect on LDL-C metabolism. Many of these above-mentioned mechanisms of action require clinical studies to be confirmed. In the future, patients could benefit from the monitoring of PCSK9 levels in serum because of the connection between platelet activation and endothelial dysfunction. The potential for expanding the indications for this new therapeutic class and clarifying their potential role in the treatment of the acute phase of ischemic cardiovascular disease may result from a thorough disentanglement of the potential pleiotropic activities of PCSK9 inhibitors, particularly of their potential anti-thrombotic effects.

## Figures and Tables

**Table 1:** Studies dealing with the non-lipid effect of PCSK9 and PCSK9 inhibitors.

Study/Authors	Number of Patients and Their Characteristics	Treatment	Result
Li et al., 2015 [49]	330, with CAD	N/A	PCSK9 levels are positively associated with the platelet count and plateletcrit, while no correlation with MPV and PDW
Pastori et al., 2017 [50]	907, with atrial fibrillation	N/A	PCSK9 levels are connected to increased risk of cardiovascular events, PCSK9 levels are correlating with thromboxane B2 levels
PCSK9—REACT study, 2017 [31]	178, with acute coronary syndrome	N/A	Increased PCSK9 levels are associated with higher platelet reactivity
Elseweidy et al. [35]	Animal model	Policosanol, 10-dehydrogingerdione	Decreased PCSK9, platelet activation and inflammation markers (sCD40L, sP-selectin, interferon-gamma)
Elseweidy et al. [51]	Animal model	10-dehydrogingerdione	Decreased interferon-gamma, sCD40L, sP-selectin correlated with PCSK9 suppression
EVOPACS study, 2018 [52]	308	PCSK9 inhibitors	Plasma levels of PCSK9 correlate with increased platelet count and platelet activation
Barale et al., 2020 [48]	24	PCSK9 inhibitors	Platelet activation markers—sCD40L, platelet factor 4, and sP-selectin, significantly decreased after treatment with PCSK9 inhibitors. These markers correlate with PCSK9.
Cammisotto et al., 2020 [34]	88, with atrial fibrillation	N/A	Markers of platelet activation and oxidative stress correlate, and changes were amplified by adding LDL
Cammisotto et al., 2021 [33]	80, heterozygous familial hypercholesterolemia	PCSK9 inhibitors	Treatment reduces platelet activation modulating NOX2 activity and, in turn, ox-LDL formation
Qi et al., 2021 [32]	N/A	PCSK9 inhibitors	PCSK9 in plasma enhances platelet activation and thrombosis by binding to CD36. PCSK9 inhibitors or aspirin abolish the enhancing effects of PCSK9, supporting the use of aspirin in patients with a high plasma level of PCSK9.
Di Minno et al., 2021 [53]	25, with familial hypercholesterolemia	PCSK9 inhibitors	Reduction in platelet-activating factors after treatment.
Marques et al., 2022 [54]	14, with familial hypercholesterolemia	PCSK9 inhibitors	PCSK9 inhibition impairs systemic inflammation and endothelial dysfunction by constraining leukocyte-endothelium interactions

## Data Availability

Data sharing not applicable.
